# Studying the Mechanobiology of Aortic Endothelial Cells Under Cyclic Stretch Using a Modular 3D Printed System

**DOI:** 10.3389/fbioe.2021.791116

**Published:** 2021-12-09

**Authors:** Sergio Aguilera Suarez, Nadia Chandra Sekar, Ngan Nguyen, Austin Lai, Peter Thurgood, Ying Zhou, Scott Needham, Elena Pirogova, Khashayar Khoshmanesh, Sara Baratchi

**Affiliations:** ^1^ School of Engineering, RMIT University, Melbourne, VIC, Australia; ^2^ School of Health and Biomedical Sciences, RMIT University, Melbourne, VIC, Australia; ^3^ Leading Technology Group, Melbourne, VIC, Australia

**Keywords:** mechanobiology, endothelial, cyclic stretch, 3D printing, mechanotransduction, biomechanics

## Abstract

Here, we describe a motorized cam-driven system for the cyclic stretch of aortic endothelial cells. Our modular design allows for generating customized spatiotemporal stretch profiles by varying the profile and size of 3D printed cam and follower elements. The system is controllable, compact, inexpensive, and amenable for parallelization and long-term experiments. Experiments using human aortic endothelial cells show significant changes in the cytoskeletal structure and morphology of cells following exposure to 5 and 10% cyclic stretch over 9 and 16 h. The system provides upportunities for exploring the complex molecular and cellular processes governing the response of mechanosensitive cells under cyclic stretch.

## Introduction

Hemodynamic forces play an essential role in maintaining vascular function and contribute to the development and progression of cardiovascular diseases (Hahn and Schwartz, 2009). There are two different types of hemodynamic forces that act on the vessel wall at each cardiac cycle, including the frictional force caused by flow-induced shear stress and the cyclic circumferential stretch caused by heartbeat (Hahn and Schwartz, 2009). The human vessel is a multi-layered structure composed of endothelial cells at the inner layer of the vessels in contact with blood flow, smooth muscle cells at the middle layer and fibroblasts at the outer layer. The effect of shear stress on endothelial cells has been extensively studied using microfluidic technologies. Such devices allow for generating customized physiological and pathophysiological flow conditions in miniaturized structures (Mohammed et al., 2019; Tovar-Lopez et al., 2019; Nguyen et al., 2021). In comparison, the effect of cyclic stretch on vascular smooth muscle cells (Mann et al., 2012; Yan et al., 2020) and fibroblasts (Sniadecki et al., 2007; Kamble et al., 2017; Kamble et al., 2018) has been widely investigated using flow-free cell stretch systems. However, the effect of cyclic stretch on endothelial cells has not been studied in detail (Estrada et al., 2011; Jufri et al., 2015).

A close look at the literature reveals a variety of flow-free cell stretch systems (Yadav et al., 2021). This includes uni-axial stretch systems, in which cells are cultured onto a rectangular elastomeric membrane. One side of the membrane is fixed while the other side is stretched using a motor-driven frame (Jungbauer et al., 2008). Several membranes can be mounted onto the frame for parallel experiments. The rectangular membrane can also be stretched from both sides to create a bi-axial stretch system (Laurence et al., 2019). Radial stretch systems are also available, in which a circular membrane is stretched radially via multiple pins positioned on the periphery of the membrane (Rápalo et al., 2015; Schürmann et al., 2016). Iris-like stretch systems have also been developed, in which a circular membrane is stretched using multiple blades that slide along both tangential and radial axes similar to aperture blades (Majd et al., 2009; Friedrich et al., 2019). Pneumatically driven radial cell stretch systems have also been demonstrated (Mann et al., 2012).

The elastomeric membrane can also be stretched using an indenter, which can be vertically displaced to push the membrane upward (Huang et al., 2010). Tailored stretch patterns can be produced by varying the profile of the indenter. Multiple membranes can also be stretched using an array of piezoelectrically actuated Braille pins (Kamotani et al., 2008), pneumatically using an automated pressure controller (Kamble et al., 2018), or hydraulically (Wang et al., 2018).

We envisage that the ability to rapidly prototype cell stretch systems using widely accessible 3D printing technologies (Bhattacharjee et al., 2016; Waheed et al., 2016) provides unprecedented opportunities to fabricate customized devices for unlocking the complex mechanobiology of endothelial cells.

In this work, we take advantage of 3D printing and soft lithography techniques to develop an inexpensive, modular, and controllable system for the cyclic stretch of aortic endothelial cells. The system utilizes cell culture chambers with soft, elastomeric membranes and a motorized cam-follower mechanism for cyclic deformation of the membrane. Customized spatiotemporal stretch profiles can be generated by varying the profile, size, and rotational speed of the cam and the follower profile. Our modular design enables the cam or followers to be changed easily. The system is mechanically robust and suitable for long-term experiments, following which the cell culture chambers can be detached from the system and interfaced with confocal fluorescence microscopy. Our experiments show the suitability of the system for studying changes in the cytoskeletal structure and morphology of human aortic endothelial cells in response to cyclic stretch.

## Principles of the System

**FIGURE 1:**
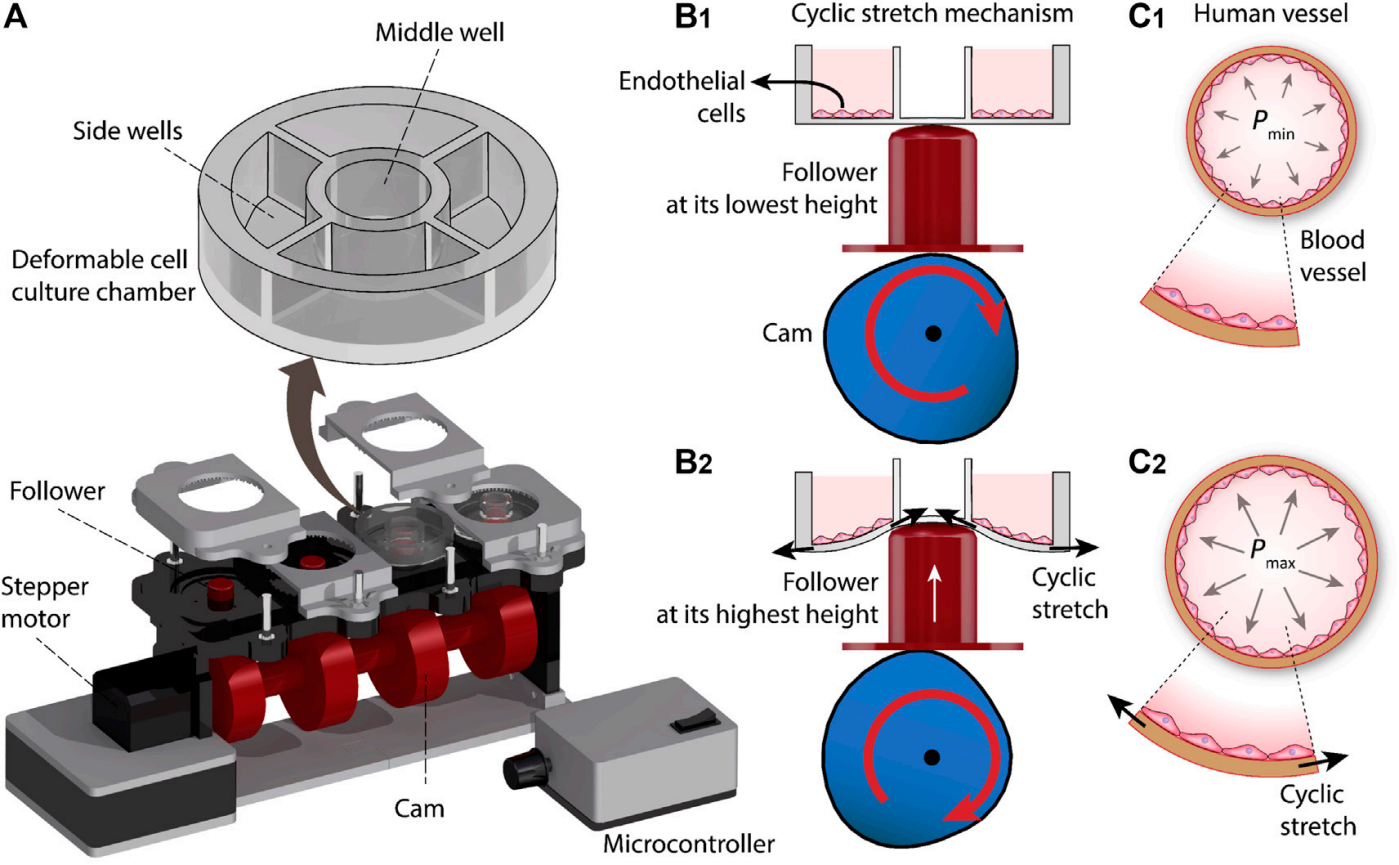
Principles of our cyclic stretch mechanism: **(A)** Schematics of the system. **(B)** Axial displacement generated by the cam during three revolutions. **(C)** Deformation of the cell culture chamber during a revolution, inducing cyclic stretch on the cultured cells.

The cyclic stretch mechanism consists of three major elements, including a deformable cell culture chamber, a follower for cyclic deformation of the cell culture chamber, and a cam for axial movement of the follower. The cam is connected to a stepper motor (Nema 17 Bipolar 59Ncm, Stepper Online) and is controlled with a microcontroller (Arduino Nano, ATmega 328). The system is equipped with four sets of cams and followers, enabling 4 cell culture chambers to be operated simultaneously (Figure 1A).

Each cell culture chamber is divided into one middle well and four side wells. The cyclic vertical displacement of the follower leads to the cyclic stretch of the middle well, which in turn is translated into the cyclic radial stretch of the side wells and the endothelial cells cultured onto them (Figure 1B). The cyclic stretch of the side wells resembles the tangential stretch of the blood vessel walls caused by cardiac pulsatile pressure (Figure 1C).

The system is equipped with 16 side wells, and thus enables 16 experiments to be conducted in parallel. The operation of the system is presented in Supplementary Video S1. The photographs of the assembled cyclic stretch system along with the detailed geometrical specifications of the system are presented in Supplementary Figures S1,2.

The system enables inducing customized cyclic stress profiles on the cultured cells by simply changing the profile, dimension, and rotational speed of the cam as well as varying the profile of the follower, as presented in Supplementary Figures S3–S7.

## Materials and Methods

### Fabrication

**FIGURE 2:**
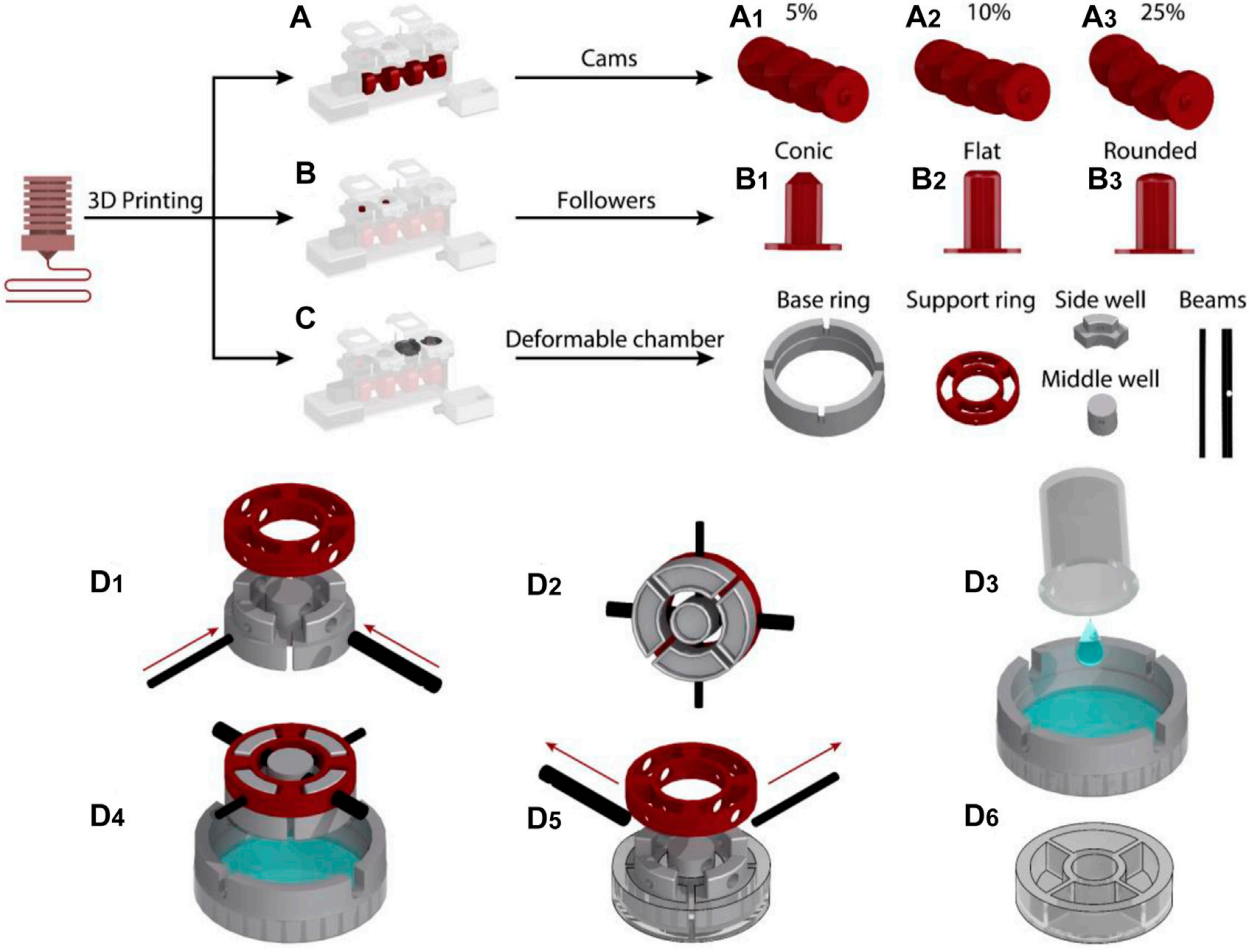
Fabrication process of the cyclic stretch mechanism: **(A)** Cam designs were 3D printed to induce 5, 10 and 25% cyclic stretch. **(B)** Followers with conic, round and flat profiles were 3D printed. **(C)** Side well, middle well, beams, support ring and base ring mold pieces to fabricate the deformable cell culture chamber were 3D printed. **(D)** Steps for the fabrication of the deformable cell culture chamber.

The cams, followers, and the chassis of the device were designed in CATIA (Dassault Systems) and 3D printed (Creality Ender 5) using polylactic acid (PLA) (PLA+, 3DFilies) (Figures 2A,B). The chassis had a modular structure so that the different components of the system could be easily joined with screws.

The deformable cell culture chamber was templated in PDMS elastomer using five sets of molds. This included the molds for side wells, middle well, beams, supporting ring and base ring, which were 3D printed with PLA (Figure 2C). The photographs of 3D printed molds are presented in Supplementary Figure S8. The side and middle well molds were interlocked to the supporting ring with two beams (Figure 2D1,2). The base ring was placed on top of a 65 mm Petri dish (Bacto Laboratories). The PDMS mixture was prepared by adding PDMS base and curing agent (Sylgard 184 Silicone elastomer kit) at a weight ratio of 10:1. The PDMS mixture was degassed and poured onto the Petri dish (Figure 2D3). The vertical grooves patterned along the sidewall of the base ring enabled the interlocked side and middle well molds to be fixed to the base ring. The vertical grooves also enabled us to set the thickness of the elastomeric membrane to 0.7 mm, ensuring that the membrane can be cyclically stretched and the cells can be visualized using an inverted microscope following long-term cyclic stretch stimulation.

The molds designed for casting of the middle and side wells incorporated an indentation with a depth of 4 mm at their lower surface facing the PDMS (Supplementary Figure S8). A small air pocket trapped inside the indentations avoided a direct contact between the PDMS and the mold surface, enabling the fabrication of cell culture chambers with a smooth surface (Supplementary Figure S9).

The PDMS was left to cure at ambient temperature for 48 h (Figure 2D4). The supporting and base rings were separated from the PDMS by removing the interlocking beams (Figure 2D5). A spatula was then used to gently separate the side and middle well molds from the cured cell culture chamber (Figure 2D6). The fabricated elastomeric cell culture chambers along with the geometrical details of the chambers are presented in Supplementary Figures S9, S10. The PDMS cell culture chambers were disposed after each round of experiments. We did not notice any fracture or structural damage in the chambers following long-term (16 h) cyclic stretch stimulation.

### Preparation of PDMS Well, Cell Culture and Cyclic Stretch System

The cell culture chamber was boiled in Milli-Q water for 1 h to improve the cellular adhesion to the cured PDMS (Park et al., 2012) and then left to cool at 4°C for 24 h. The wells were then UV-treated for 10 min to be sterilized, coated with fibronectin (50 μg/ml) and stored at 4°C for 24 h. Primary human aortic endothelial cells (HAECs) were purchased from Lonza and cultured in EGM-2 media supplemented with a SingleQuots kit according to the supplier’s instructions. Cells were incubated at 37°C in a humidified incubator and 5% CO_2_. HAECs were passaged every 2–3 days and used for up to 5 passages. For cyclic stretch experiments, HAECs were cultured inside the fibronectin-coated cell culture chambers at 37°C for 24 h so that the endothelial cells would be able to adhere to the bottom surface of the wells. The chambers were then loaded onto the device and subjected to 5 and 10% cyclic stretch at 1 Hz for varying timeframes (1, 3, 9 and 16 h) at 37°C. Static wells were just left in the incubator at 37°C for the same timeframes.

### Immunocytochemistry and Confocal Microscopy

Upon removing the cell culture chambers from the device, cells were rinsed in phosphate-buffered saline (PBS) twice and fixed in 4% paraformaldehyde at 37°C for 1 h. The cells were subsequently washed twice in PBS and then blocked with 5% goat serum at 37°C for 1 h to avoid non-specific antibody binding. Next, F-actin was stained with Atto 565-phalloidin (Sigma-Aldrich, 1/400 dilution) and nuclei were stained with DAPI (Thermo Scientific, 1/400 dilution). All wells were incubated at 37°C for 2 h, after which they were rinsed four times with PBS. Image acquisition for all experiments was performed with a Nikon A1MP Multiphoton microscope controlled by Nikon Elements software (Nikon).

### Image Analysis

Image processing of actin filaments were performed using ImageJ and NIS Elements software (Nikon). The orientation of the cells and stress fibers was determined by an automated image processing algorithm written in MATLAB, as explained in the Supplementary Information S8.

Image processing of nuclear area and circularity were performed using ImageJ and NIS Elements software (Nikon). The analysis of nuclear area and circularity was determined by an automated image processing algorithm written in MATLAB, as explained in the Supplementary Information S8.

## Results and Discussions

### Cyclic Stretch Controls the Orientation of Actin Filaments

**FIGURE 3:**
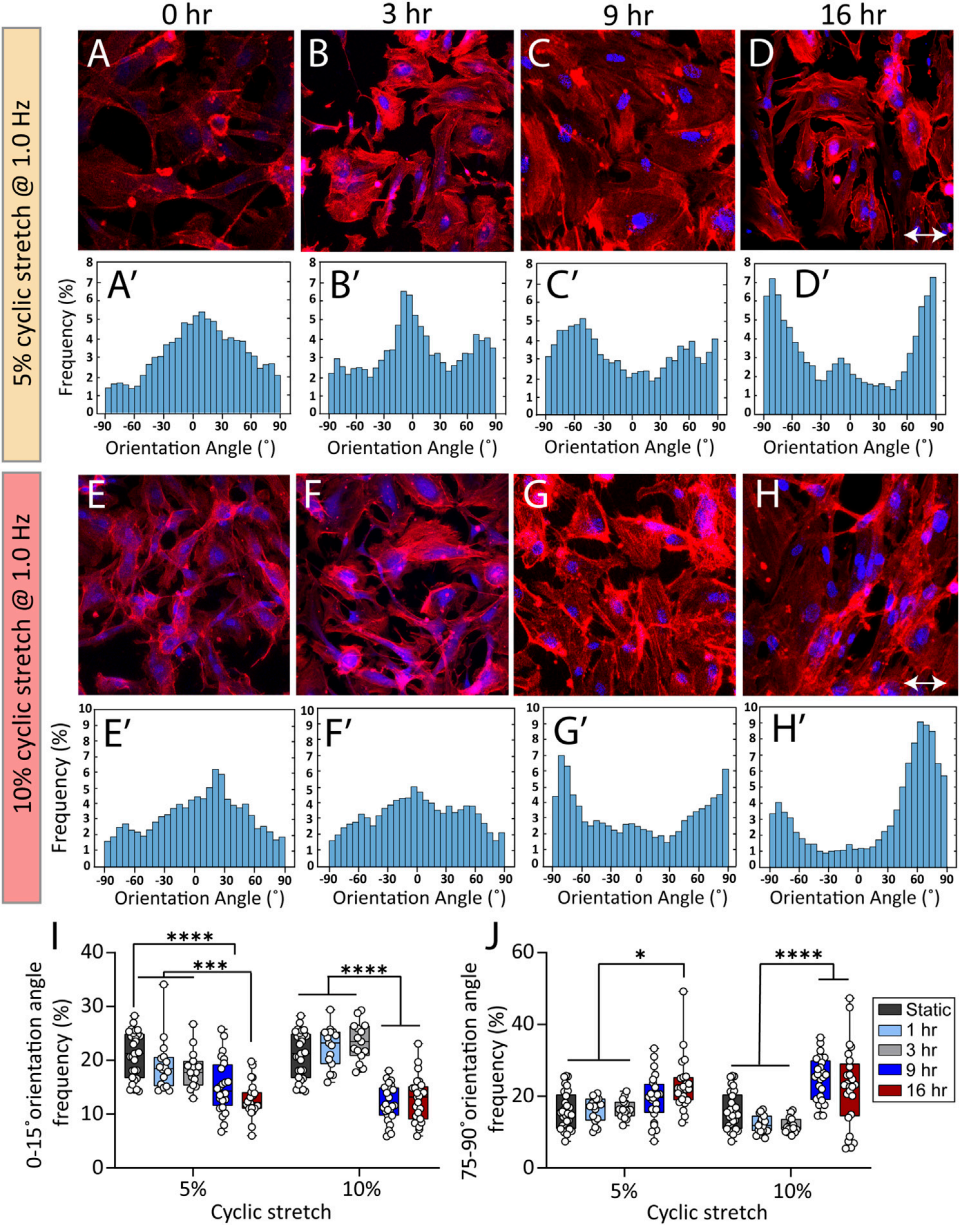
Cyclic stretch controls the orientation of stress fibers in HAECs. **(A–H)** Immunofluorescent images of actin stress fibers in HAECs cultured under static and cyclic stretch conditions under cyclic stretch levels of 5 and 10%. Actin is labelled with Atto 565-phalloidin (red) and the nucleus is labelled with DAPI (blue). Double-headed arrows indicate the stretch direction. **(Aʹ–Hʹ)** Histograms showing the frequency of stress fibres orientation angle at different stretch levels and exposure periods. **(I,J)** Summary graphs comparing the frequency of HAECs stress fibres with orientation angles of **(I)** 0–15° and **(J)** 75°–90° when subjected to cyclic stretch levels of 5 and 10% under various exposure periods. Circles represent single cells. Boxes show median and first and third quartiles. *p < 0.05, ***p < 0.001, ****p < 0.0001, two-way ANOVA and Tukey’s multiple comparisons test. Cyclic stretch controls the morphology of endothelial cells and the cell nucleus.

The cyclic strectch imposed by cardiac cycle exerts a dynamic force to the cell membrane, focal adhesions, cytoskeletal filaments, and the nucleus (Walker et al., 2020). We harnessed the versatility of our system to study the effect of cyclic stretch on the orientation of actin filaments at different exposure periods. To do so, endothelial cells were cultured inside the side wells, which were precoated with fibronectin for 24 h, allowing them to adhere and form a monolayer. The side wells were stretched using cam profiles capable of generating dynamic trapezoidal displacement profiles coupled to flat followers. The orientation angle of stress fibres was assessed at different conditions of control (static), 5 and 10% cyclic stretch at 1 Hz following exposure to cyclic stretch for 3, 9, and 16 h (Figures 3A–H). The response of cells after 1 h along with psedu-colored contours showing the orientation of stress fibers are presented in Supplementary Figure S11. We did not observe any significant difference in the morphology and viability of endothelial cells under static condition at different time points.

Figures 3I,J compare the frequency of actin filaments with orientation angles of 0°–15° and 75°–90° under cyclic stretch levels of 5 and 10% obtained after 1, 3, 9, and 16 h of exposure.

Under static conditions, actin filaments were randomly oriented (Figure 3A-Aʹ). Exposure of cells to 5% cyclic stretch for 1, 3, and 9 h, did not significantly affect the orientation angle of stress fibres compared to static conditions (Figure 3B-Bʹ and Figure 3C-Cʹ). In comparison, exposure of cells to 5% cyclic stretch for 16 h, decreased the percentage of actin filaments aligned at an orientation angle of 0°–15° from 21.2 ± 0.7 under static conditions to 12.7 ± 0.6 after 16 h (Figure 3D-Dʹ). Correspondingly, the percentage of actin filaments with an orientation angle of 75°–90° increased from 16.3 ± 1.4 under static conditions to 23.2 ± 1.5 after 16 h (Figure 3D-Dʹ).

Similarly, exposure of cells to 10% cyclic stretch did not significantly change the orientation angle of actin stress fibres following 1 and 3 h of exposure compared to static conditions (Figure 3F-Fʹ). However, longer exposure of 9 and 16 h decreased the percentage of actin filaments with an orientation angle of 0°–15° from 21.2 ± 0.7 under static conditions to 12.5 ± 0.6 and 12.7 ± 0.8 after 9 and 16 h, respectively (Figure 3G-Gʹ and Figure 3H-Hʹ). Correspondingly, the percentage of actin filaments displaying an orientation angle of 75°–90° increased from 16.3 ± 1.4 under static conditions to 24.7 ± 1.2 and 22.6 ± 2.2 after 9 and 16 h, respectively.

The changes in the orientation of actin filaments caused by a 10% cyclic stretch for 9 h was similar to the changes caused by a 5% cyclic stretch for 16 h, suggesting that changes in the cytoskeletal structure depends on both the magnitude and duration of the cyclic stretch. Our findings are in line with previous observations showing the reorientation of actin cytoskeleton perpendicular to stretch direction as a mechanism to maintain cell structure and integrity by reducing the strain and tension on the cell (Liu et al., 2008; Livne et al., 2014).

### Cyclic Stretch Controls the Morphology of Endothelial Cells and the Cell Nucleus

**FIGURE 4:**
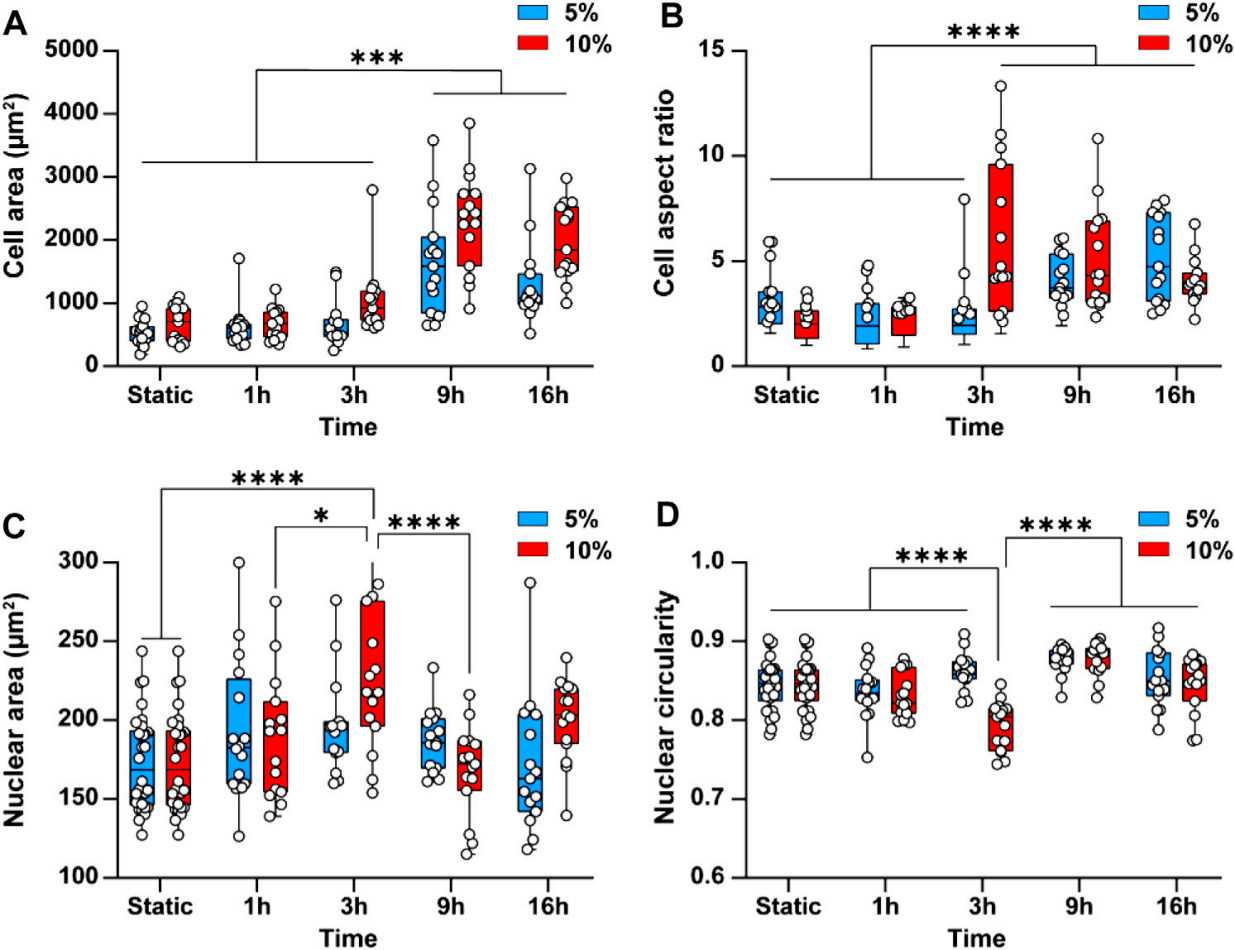
Cyclic stretch changes the morphology of endothelial cells and the cell nucleus. Bar graphs showing the differences in **(A)** Cell area and **(B)** Cell aspect ratio **(C)** Average nuclear area and **(D)** Average nuclear circularity when subjecting cells to 5 and 10% cyclic stretch for different exposure periods. Circles represent single cells. Boxes show median and first and third quartiles. * indicates *p* < 0.05, ** indicates *p* < 0.01, *** indicates *p* < 0.001 and **** indicates *p* < 0.0001.

Cyclic stretch is shown to increase cell spreading and elongation, thus changing the morphology of the cells (Yamada et al., 2000). This inspired us to examine the effect of cyclic stretch on the average area and aspect ratio of endothelial cells. Endothelial cells not exposed to cyclic stretch (static conditions) had a rounded polygonal shape with an average area of 602 ± 127 µm^2^. Exposure of cells to 5 and 10% cyclic stretch caused cell spreading and increased the cell area by 2.7 ± 1 and 3.8 ± 0.5 folds, respectively, after 9 h (Figure 4A). Likewise, the cell aspect ratio increased by 2 ± 0.6 folds after 16 h (*p* < 0.001) under 5% cyclic stretch and by 2.4 ± 0.5 folds after 3 h (*p* < 0.001) under 10% cyclic stretch (Figure 4B). This is in line with previous results reporting the effect of cyclic stretch magnitude on the morphology of endothelial cells (Hahn and Schwartz, 2009).

Cyclic stretch has been found to change the cell nucleus shape and size (Heo et al., 2015; Seelbinder et al., 2020). This inspired us to investigate the effect of cyclic stretch on the nucleus area and circularity. The cell nucleus was stained with DAPI and measured nuclear area and circularity changes after different exposure periods. Applying a 5% cyclic stretch did not cause any significant differences in average nuclear area and circularity (Figures 4C,D). However, applying a 10% cyclic stretch increased the nuclear area by 1.3 ± 0.26 folds (*p* < 0.001) and conversely decreased the nuclei circularity by 1 ± 0.2 folds (*p* < 0.001) after 3 h. These findings indicate that change in morphology of endothelial cells depends on the magnitude and duration of the cyclic stretch similar to what we observed in the cytoskeletal structure. Both nuclear area and circularity returned to their basal level after 9 h (Figures 4C,D). This behaviour can be attributed to the mechanisms developed by the cell nucleus to robustly function in the presence of mechanical stress to prevent the nuclear rupture (Polychronidou and Grobhans, 2011). For example, the nuclear envelope lamina network provides force dampening and act as a ‘molecular shock absorber’ (Dahl et al., 2004). In addition, the nucleus is a dynamic structure and adjusts its stiffness to resist mechanical stress through the phosphorylation of emerin protein present at the inner nuclear membrane (Guilluy et al., 2014). Owing to these mechanisms, the nuclear swelling is usually nonlinear.

## Conclusion

In summary, we demonstrated a cam-driven system for cyclic stretch of aortic endothelial cells. We harnessed the versatility of the system for studying the cytoskeletal structure and morphology of aortic endothelial cells in response to cyclic stretch. Using a combination of fluorescent microscopy and image processing approach, we showed that cyclic stretch leads to the perpendicular alignment of the endothelial actin stress fibres, increases the cell area and aspect ratio in a dose- and time-dependent manner. Furthermore, we found that exposure of cells to 10% cyclic stretch increases the nuclear area while reduces its circularity within the first 3 h but returned to basal levels after 16 h. The controllability and simplicity of the system make it suitable for exploring the mechanobiology of various cells under cyclic stretch.

## Data Availability

The original contributions presented in the study are included in the article/Supplementary Material, further inquiries can be directed to the corresponding authors.
