# Supplementary material for: Studying the Mechanobiology of Aortic Endothelial Cells Under Cyclic Stretch Using a Modular 3D Printed System
Source: Front Bioeng Biotechnol. 2021 Dec 9;9:791116. doi: 10.3389/fbioe.2021.791116 (PMC8698250; doi:10.3389/fbioe.2021.791116)
Supplement: Supplementary file 1 [file Presentation1.pdf]

# Supplementary Information

# Studying the mechanobiology of aortic endothelial cells under cyclic stretch using a modular 3D printed system

Sergio Aguilera Suarez [1, *], Nadia Chandra Sekar [2,*], Ngan Nguyen [1], Austin Lai [2],

Peter Thurgood [1], Ying Zhou [2], Scott Needham [3], Elena Pirogova [1],

Khashayar Khoshmanesh [1,**, †], Sara Baratchi [2,**,†]

[1] School of Engineering, RMIT University, Victoria 3000, Australia

[2] School of Health and Biomedical Sciences, RMIT University, Victoria 3083, Australia

[3] Leading Technology Group, Victoria 3153, Australia

* and ** These authors contributed equally to this work.

**† Corresponding authors:**

Khashayar.Khoshmanesh@rmit.edu.au

Sara.Baratchi@rmit.edu.au

**Supplementary Information 1:** 3D printed cyclic stretch mechanism

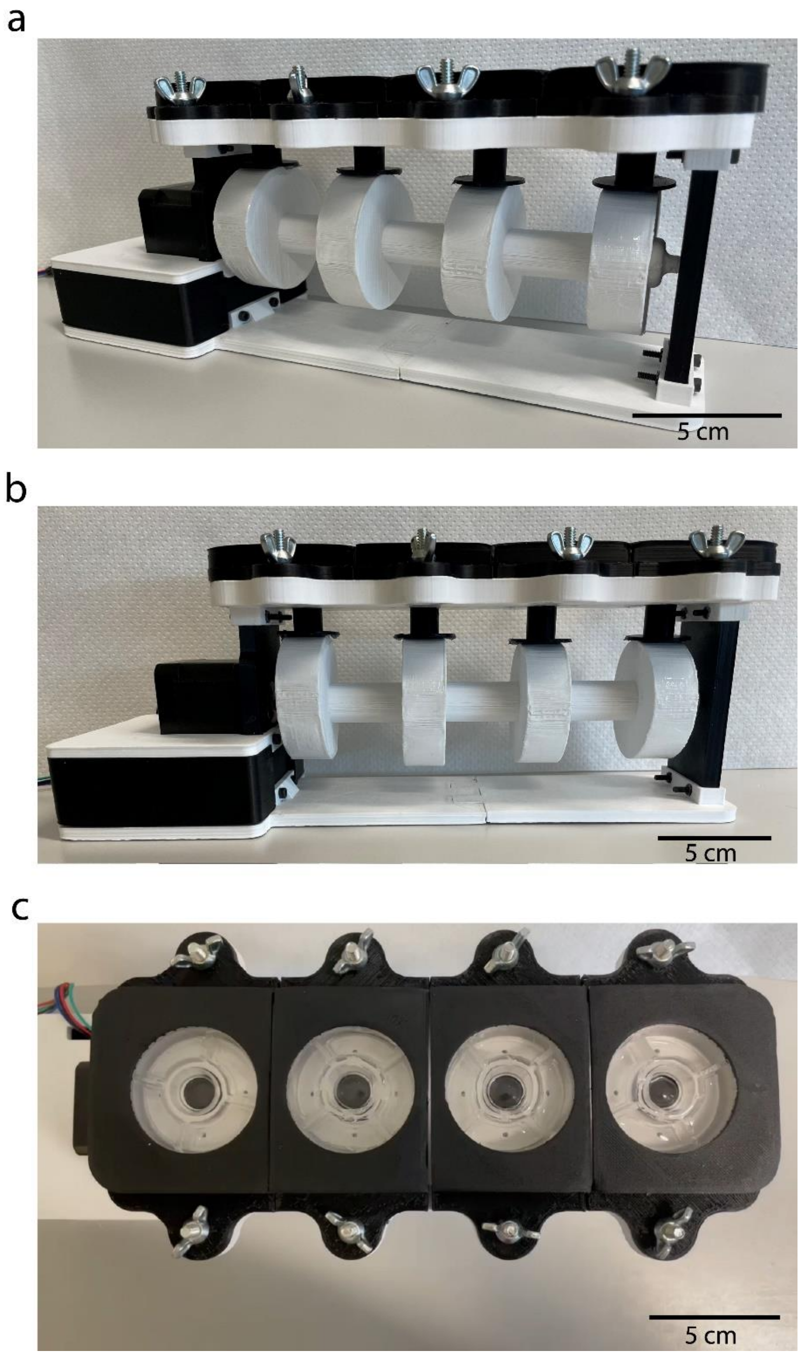


**Figure S1.** Photographs of the 3D printed cyclic cell stretch mechanism: **(a)** Isometric view. **(b)** Side view. **(c)** Top view.

**Supplementary Information 2:** Geometrical details of the cyclic cell stretch mechanism

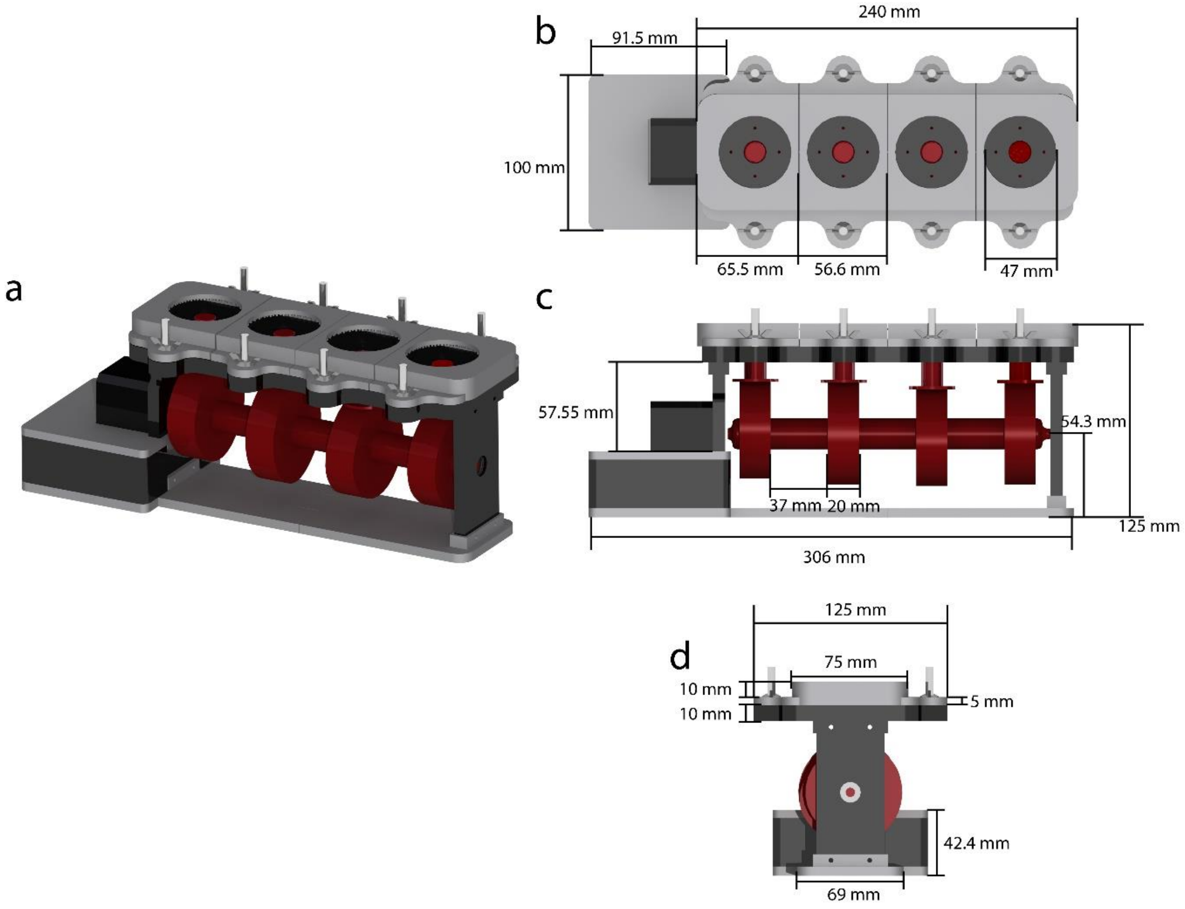


**Figure S2.** Detailed dimensions of the cyclic cell stretch mechanism: **(b)** Isometric view. **(b)** Top view. **(c)** Side view. **(d)** Front view.

**Supplementary Information 3:** Characterisation of the cyclic stretch system

The versatility of our cyclic stretch system enabled us to generate tailored spatiotemporal strain/stress profiles by changing the cam profile, cam size, and follower profile (**Figure S3a**). To showcase this versatility, we designed three cam profiles to generate cyclic trapezoidal, sine, and double-peak displacement profiles (**Figure S3b**). The cams were designed in Matlab using cycloidal motion cam dynamics (Charles E. Wilson, 2003; Norton, 2005), as detailed in **Supplementary Information 4**.

The dimensions of the cams were varied to generate relative vertical displacements (defined as the ratio of axial displacement to the diameter of the cell culture chamber, which is referred to as cyclic stretch) of 5%, 10%, 15%, 20%, and 25% (**Figure S3b**). We also designed three followers with conic, rounded, and flat profiles (**Figure S3c**).

Numerical simulations were performed using ANSYS static structural module to predict the strain/stress profile of the membrane placed at the bottom surface of the cell culture chamber under various cam/follower combinations. In the first set of simulations, we investigated the dynamic variations of strain/stress at the middle section of the side well membrane (**Figure S3a**). The membrane was displaced using cams capable of generating dynamic trapezoidal, sine, and double-peak displacement profiles when coupled to a flat follower. Our simulations indeed show the ability to generate customized spatiotemporal strain/stress profiles by varying the profile and size of the cam (**Figure S3d**).

In the second set of simulations, we investigated the maximum strain/stress induced at the middle section of the side well membrane. The membrane was displaced by coupling cams capable of generating dynamic trapezoidal displacement profiles to conic, rounded and flat followers (**Figure S3e**).

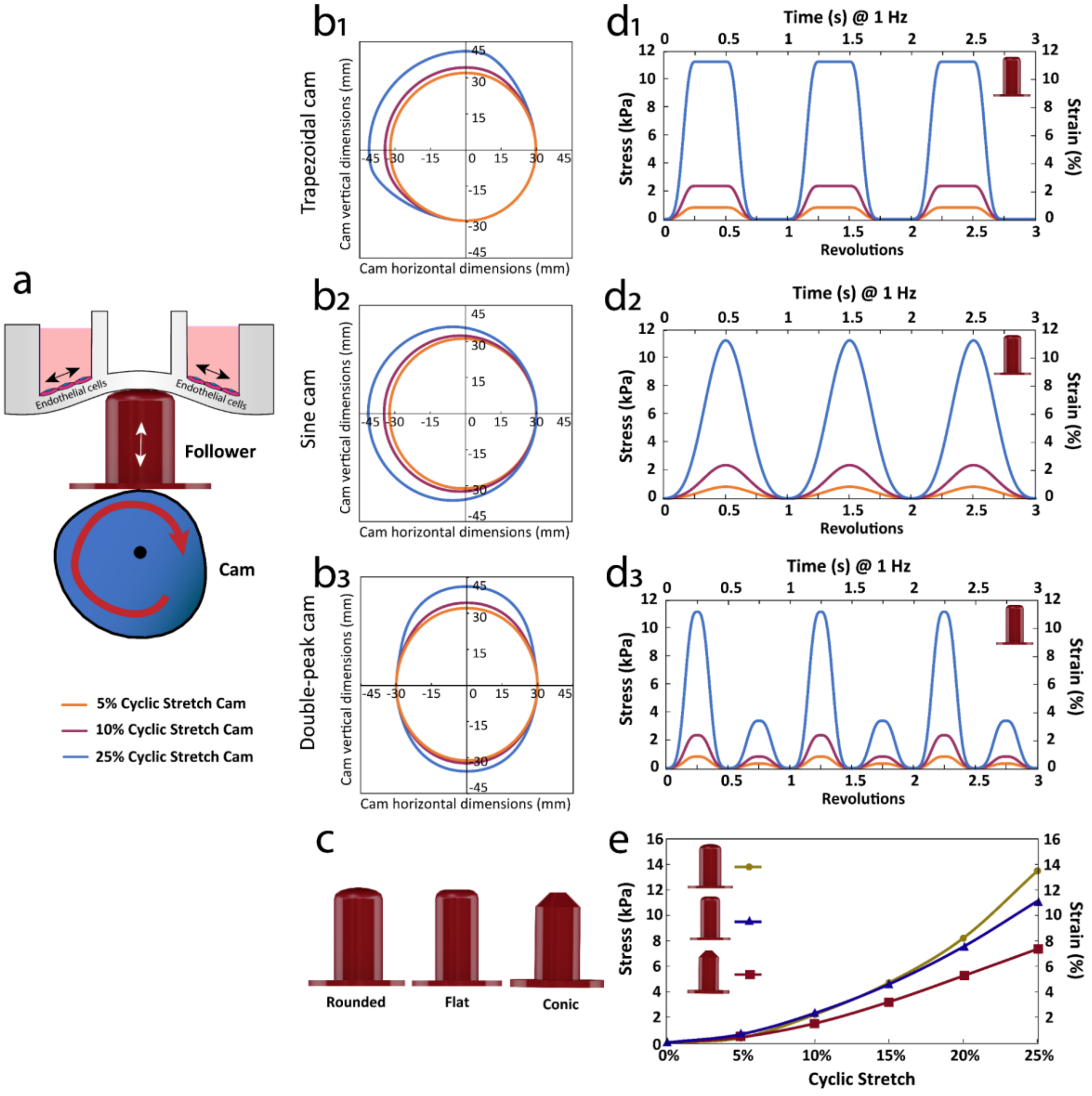


**Figure S3.** Comparing the stress induced by various follower profiles: **(a)** Schematics of the cyclic stretch system. **(b)** Cam profiles capable of generating dynamic trapezoidal, sine and double-peak displacement profiles. **(c)** Followers with conic, rounded and flat profiles. **(d)** Dynamic variation of strain/stress profile at the middle section of the side well membrane over successive cycles of cam rotation when coupling cams with various profiles and sizes to a flat follower. **(e)** Maximum strain/stress induced at the middle section of the side well membrane when coupling cams capable of generating dynamic trapezoidal displacement profiles to conic, rounded and flat followers.

**Supplementary Information 4:** Detailed cam design

The cams were designed using standard design techniques, in which a displacement motion is generated by a series of standardized equations. The cams comprised of a rising segment, where the displacement increases, and a falling segment, where the displacement decreases. Additional elements were added to the cams to customize them to our needs. These elements were dwells, which keep the displacement constant at the desired segments. The combination of rising, falling and dwell segments allowed us to generate customized motion profiles with varying magnitudes:

## A: Trapezoidal displacement design

To design the trapezoidal cam, a combination of standard cam cycloidal displacements and dwells were used where $L$ is the maximum displacement (stroke) and $\theta$ is the cam angle of rotation.

$$S(\theta) = \begin{cases} L\left(\frac{\theta}{90} - \frac{1}{2\pi} sin\ 2\pi \frac{\theta}{90}\right) & 0° \le \theta \le 90° \\ L & 90° \le \theta \le 180° \\ L\left(1 - \frac{(-180+\theta)}{90} + \frac{1}{2\pi} sin\ 2\pi \frac{(-180+\theta)}{90}\right) & 180° \le \theta \le 270° \\ 0 & 270° \le \theta \le 360° \end{cases}$$

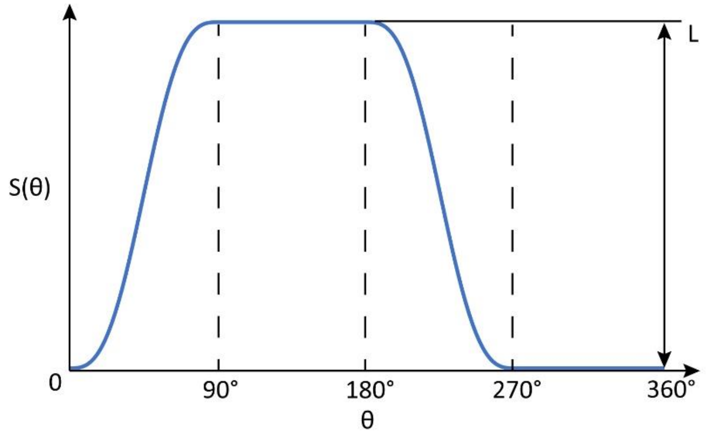


**Figure S4.** Trapezoidal displacement design: A rising cycloid motion was used within 0°-90°, followed by a dwell at the maximum displacement within 90°-180°, a falling cycloid for within 180°-270°, and a dwell within 270°-360°.

**B: Sinusoidal displacement design**

To design the sinusoidal cam, a standard harmonic displacement was used, where $L$ is the maximum displacement (stroke) and $\theta$ is the cam angle of rotation.

$$S(\theta) = \begin{cases} \frac{L}{2}\left(1 - cos\frac{\pi\theta}{180°}\right) & 0° \leq \theta \leq 180° \\ \frac{L}{2}\left(1 + cos\frac{\pi(-180 + \theta)}{180°}\right) & 180° \leq \theta \leq 360° \end{cases}$$

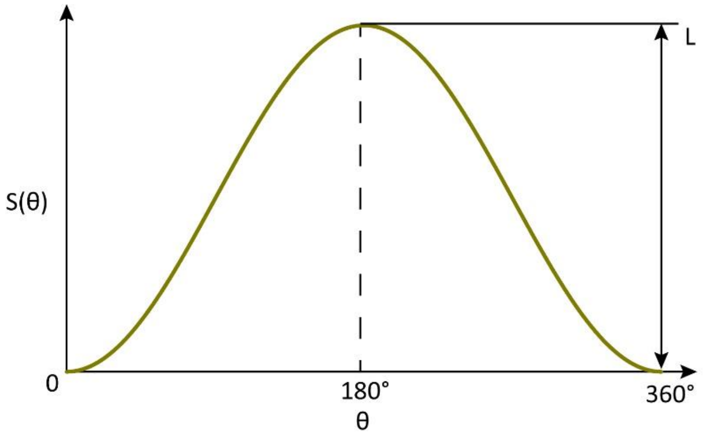


**Figure S5.** Sinusoidal displacement design: A rising harmonic motion was used within 0°-180°, followed by a falling harmonic motion within 180°-360°.

**C: Double-peak displacement design**

To design the double-peak cam, two sets of harmonic displacements were used. On the first half, the maximum displacement was set to $L$ while in the second half, the maximum displacement was halved. This arrangement generated two peaks with different magnitudes during each revolution, where $\theta$ is the cam angle of rotation.

$$S(\theta) = \begin{cases} \frac{L}{2}\left(1 - cos\frac{\pi\theta}{90}\right) & 0° \leq \theta \leq 90° \\ \frac{L}{2}\left(1 + cos\frac{\pi(-90+\theta)}{90}\right) & 90° \leq \theta \leq 180° \\ \frac{L}{4}\left(1 - cos\frac{\pi(-180+\theta)}{90}\right) & 180° \leq \theta \leq 270° \\ \frac{L}{4}\left(1 + cos\frac{\pi(-270+\theta)}{90}\right) & 270° \leq \theta \leq 360° \end{cases}$$

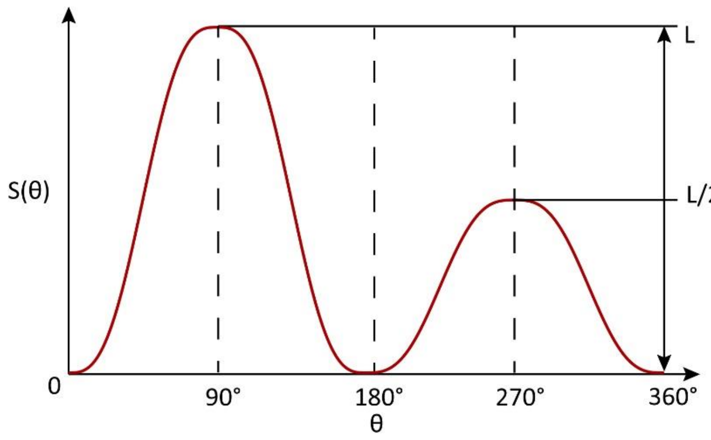


**Figure S6.** Double-peak displacement design: A rising harmonic motion was used within 0-90° of the motion, followed by a falling harmonic motion within 90°-180 with a maximum displacement of *L*. A similar trend was followed for the second half of the harmonic motion with a maximum displacement of *L/2*.

**D: Cam design equations**

The minimum cam radius $(R_o)$, and the horizontal $(x)$ and vertical $(y)$ dimensions of the cams were calculated as follows:

$$R_o = S(\theta) + S''(\theta)$$
$$x(\theta) = (R_o + S(\theta))\cos\theta - S'(\theta)\sin\theta$$
$$y(\theta) = (R_o + S(\theta))\sin\theta + S'(\theta)\cos\theta$$

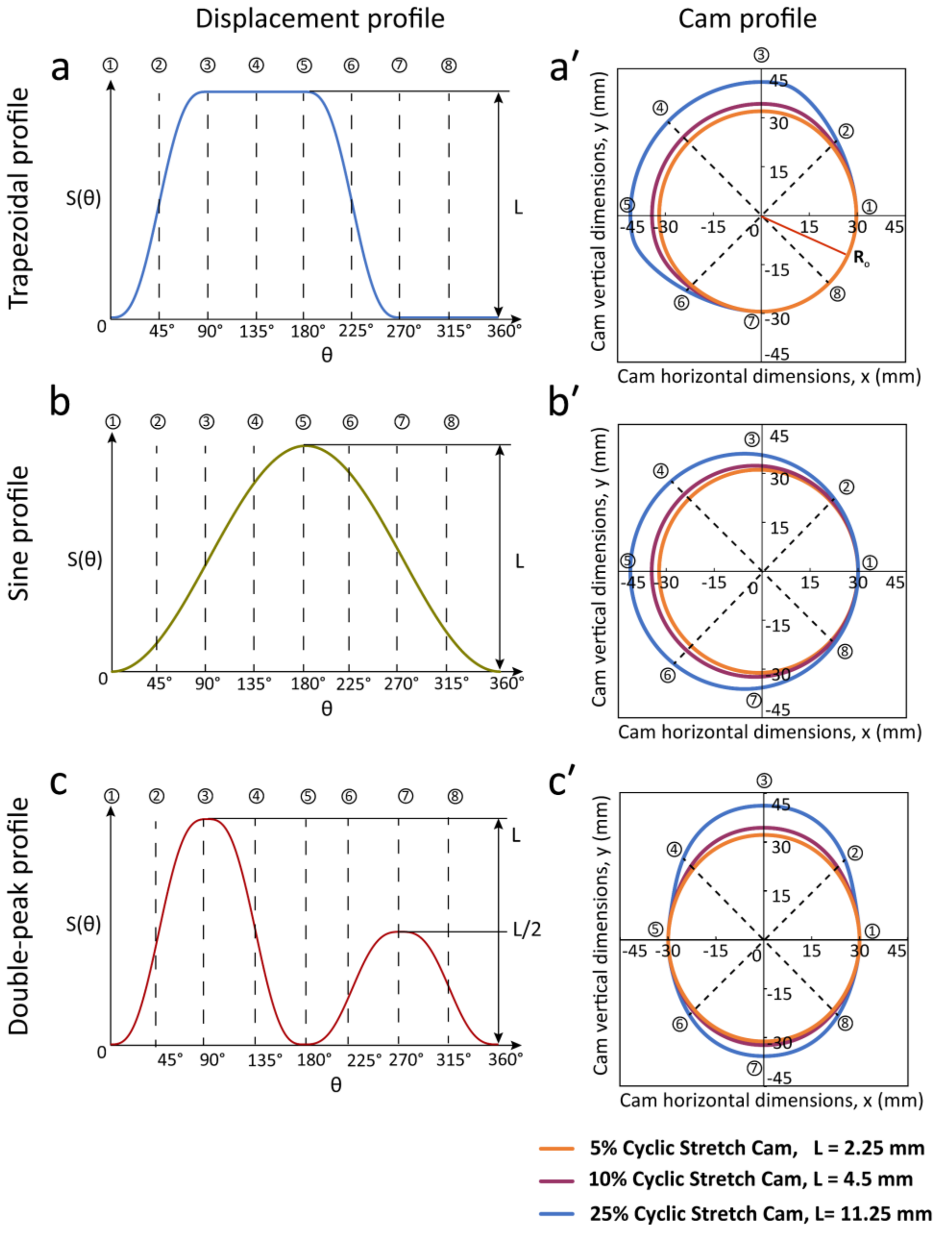


**Figure S7.** Cam design: (a-c) Displacement profiles for trapezoidal, sine and double-peak cams. (a′-c′) Generated profiles for trapezoidal, sine and double-peak cams with various strokes to induce 5%, 10% and 25% cyclic stretch.

**Supplementary Information 5:** 3D printed molds for casting of the cell culture chambers

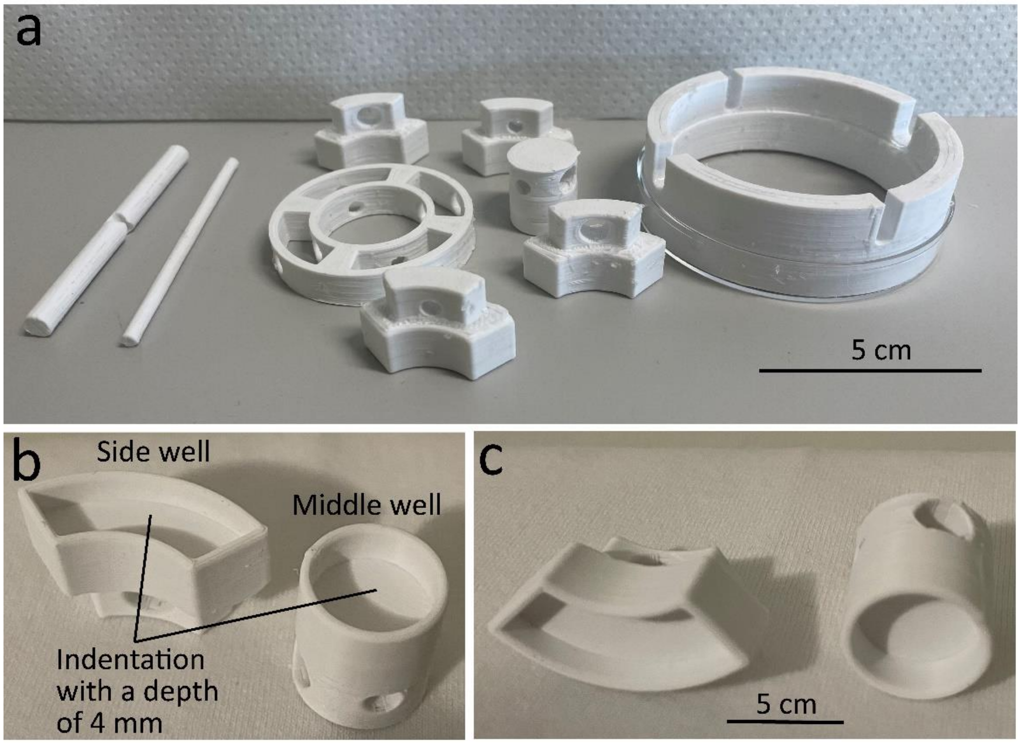


**Figure S8**. 3D printed molds for casting of the cell culture chambers: **(a)** 3D printed side well, middle well, beams, support ring and base ring mold pieces to fabricate the deformable cell culture chamber. **(b-c)** Closeup view of the side well and middle well, clearly showing the 4 mm indentation at the bottom surface of the wells to accommodate a small air pocket.

**Supplementary Information 6:** Utilization of small air pockets to fabricate smooth cell culture chambers

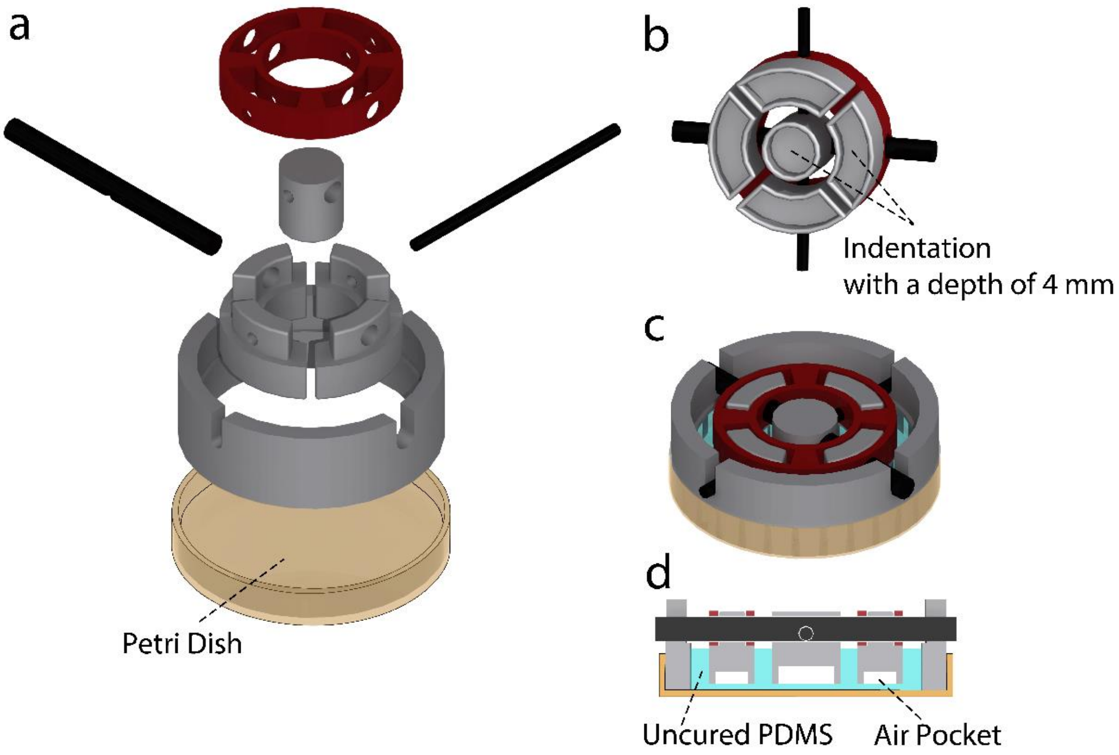


**Figure S9**. The incorporation of an indentation with a depth of 4 mm at the surface of the middle and side well molds to enable trapping a small air pocket.

**Supplementary Information 7:** Geometrical details of the cell culture chamber

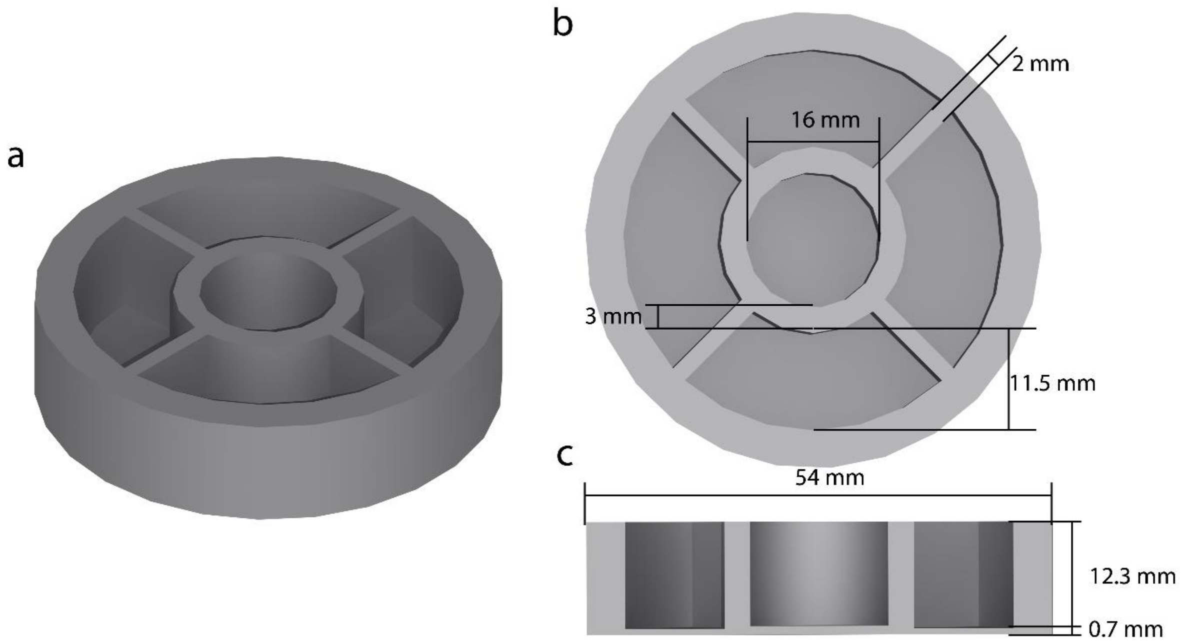


**Figure S10**. Geometrical details of the elastomeric cell culture chamber: **(a)** Isometric view. **(b)** Top view. **(c)** Side view.

**Supplementary Information 8:** Image analysis to quantify the changes in the orientation of stress fibers, cellular area, nuclear area and aspect ratio

The orientation of the cells and stress fibers was determined by an automated image processing algorithm written in MATLAB. Briefly, the algorithm computes the orientation of the actin filaments based on spatial gradients; by generating pixel-by-pixel magnitude and direction information from intensity gradients in the horizontal and vertical directions. The information was then used to construct histograms of dominant orientations for each pixel. The histograms evaluated deviation from a set of angles ranging from –89° to 90° relative to the horizontal using a small subregion (20×20 pixels), which was centred around the pixel of interest. The Statistical significance was assessed with a global Watson's U2 test, and statistics were computed using the circular statistic toolbox. For statistical analysis, one-way ANOVA was performed using Prism 8 (GraphPad software), and $P < 0.05$ was considered significant.

The analysis of nuclear area and circularity was determined by an automated image processing algorithm written in MATLAB. This algorithm uses contrast filters to create a color difference between the nucleus and the structure of the cell. Next, it binarizes the image to separate, label, and measure the number of pixels and perimeter in each nucleus. The number of pixels obtained per cell is used to calculate the area. The circularity is obtained as follows, in which $A_{cell}$ is the cell area and $P_{cell}$ is the cell perimeter:

$$Cell\ circularity = \frac{4\pi \times A_{cell}}{P_{cell}^2}$$

In which a circularity closer to 1 is rounder. The Statistical significance was assessed with a global Watson's U2 test, and statistics were computed using the circular statistic toolbox. For statistical analysis, one-way ANOVA was performed using Prism 8 (GraphPad software), and $P < 0.05$ was considered significant.

**Supplementary Information 9:** Cyclic stretch controls the orientation of stress fibers in human aortic endothelial cells (extended version of Figure 3)

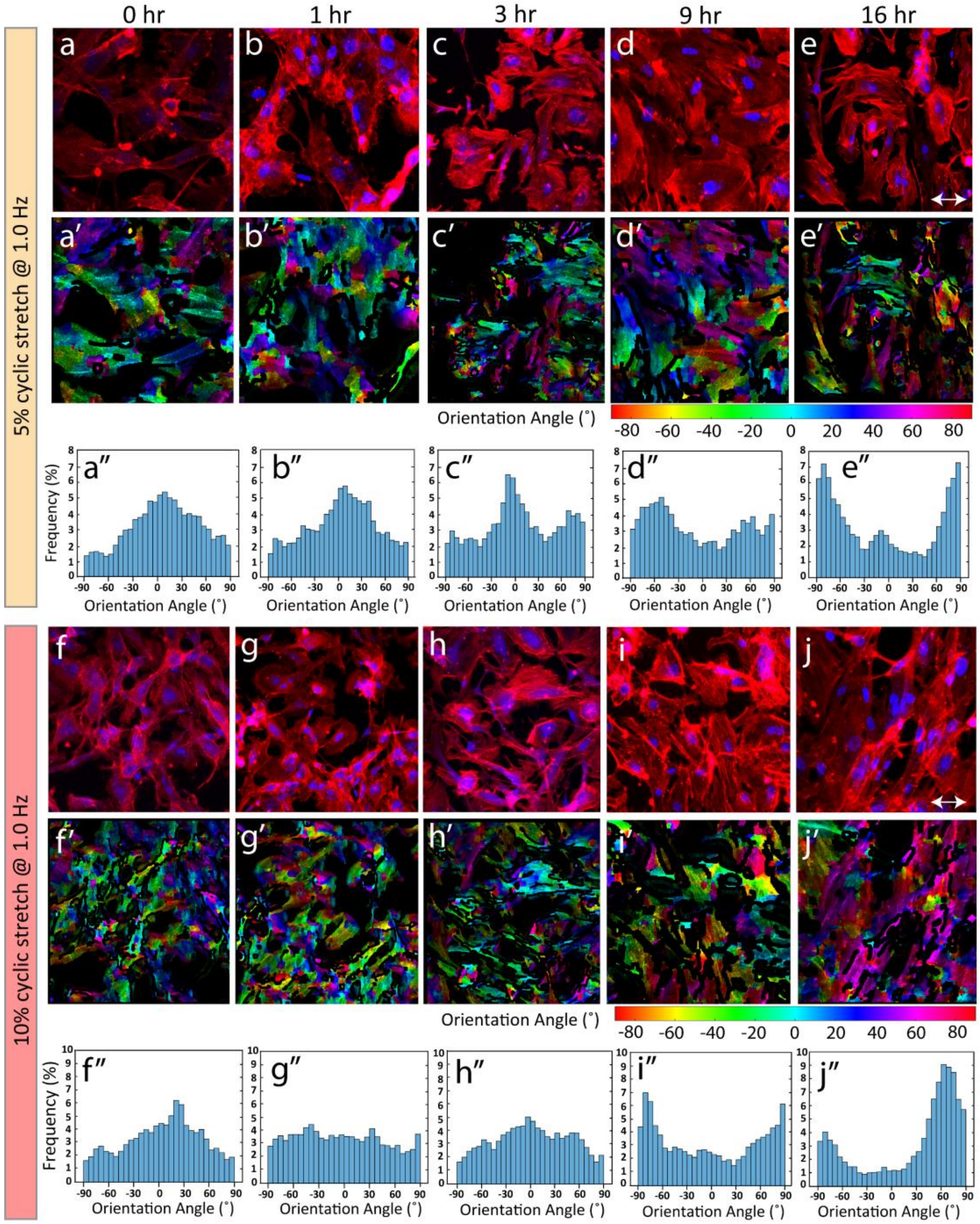


**Figure S11.** Cyclic stretch controls the orientation of stress fibers in human aortic endothelial cells (HAECs): **(a-j)** Immunofluorescent images of actin stress fibers in HAECs cultured under static and cyclic stretch levels of 5% and 10%. Double-headed arrows indicate the stretch direction. **(a′-j′)** Pseudo-colored contours displaying the orientation angle of stress fibers in HAECs. **(a″-j″)** Histograms showing the frequency of stress fibres orientation angle at different stretch levels.
